# Cesarean delivery and metabolic health and inflammation biomarkers during mid-childhood and early adolescence

**DOI:** 10.1038/s41390-021-01503-9

**Published:** 2021-04-06

**Authors:** Lidia Mínguez-Alarcón, Sheryl L. Rifas-Shiman, Caroline Mitchell, Joanne Sordillo, Izzuddin M. Aris, Marie-France Hivert, Emily Oken, Jorge E. Chavarro

**Affiliations:** 1 Department of Environmental Health, Harvard School of Public Health, Boston, MA USA; 2 Department of Nutrition, Harvard School of Public Health, Boston, MA USA; 3 Department of Population Medicine, Harvard Medical School and Harvard Pilgrim Health Care Institute, Boston, MA USA; 4 Department of Obstetrics and Gynecology, Massachusetts General Hospital, Boston, MA USA; 5 Diabetes Unit, Massachusetts General Hospital, Boston, MA USA; 6 Department of Epidemiology, Harvard School of Public Health, Boston, MA USA; 7 Department of Medicine, Harvard Medical School and Brigham and Women’s Hospital, Boston, MA USA

## Abstract

**Background:**

We assessed differences in plasma levels of metabolic health and inflammation biomarkers during mid-childhood and early adolescence between children born by cesarean section vs. vaginal delivery.

**Methods:**

Mother–child pairs (*N* = 942) enrolled during pregnancy in obstetric practices and child follow-up started at birth. Risk biomarkers were assessed in blood samples collected at the mild-childhood (median = 7 years) and early adolescence (median = 13 years) in-person visits.

**Results:**

Two hundred and six children (22%) were born by cesarean section. There were no significant differences in biomarker levels between children born by cesarean and children born vaginally in mid-childhood. However, adolescents born by cesarean section had significantly lower adiponectin [% difference (95% confidence interval (CI)) = −11.3 (−18.1, −4.0) µg/mL] compared to vaginal delivery. We also found some suggestion of higher insulin resistance [insulin levels % difference (95% CI) = 11.5 (−0.40, 25.0) µU/mL and HOMA-IR (homeostatic model assessment of insulin resistance) % difference (95% CI) = 9.1 (−2.30, 21.8) U] in adolescents born by cesarean section compared to those born vaginally.

**Conclusions:**

We found suggestive evidence that adolescents born by cesarean section show differences in certain metabolic health biomarkers relative to adolescents born by vaginal delivery. Further studies are needed to reevaluate these associations since the clinical significance of these differences is unclear.

**Impact:**

- Multiple studies show that children born by cesarean section are at higher risk of obesity compared to those born vaginally.
- It is unclear yet to what extent this elevated risk may extend to a more adverse profile of biomarkers of metabolic health and inflammation.
- Adolescents born by cesarean section show small differences in adiponectin and insulin relative to adolescents born by vaginal delivery.
- Adolescents born by cesarean section may be at higher risk to a more adverse profile of biomarkers of metabolic health and inflammation, but the clinical significance of these differences is uncertain.

## Introduction

Cesarean deliveries are the most common inpatient surgical procedure in the United States,^1^ accounting for approximately one-third of deliveries nationwide.^2,3^ In addition to the immediate risks to mother and newborn,^4–7^ increasing evidence shows that children born by cesarean section may also experience higher rates of adverse health outcomes later in life,^8^ including type 1 diabetes,^9^ allergies and asthma,^10–12^ multiple sclerosis,^13^ and chronic immune disorders.^14,15^ Although not observed in all studies,^16,17^ the vast majority,^18–21^ as well as three meta-analyses,^22–24^ have also shown a relationship between birth by cesarean delivery and higher risk of childhood obesity. We have previously reported that, compared to children born via vaginal delivery, children born by cesarean delivery have a higher body mass index (BMI)-*z* through childhood and early adolescence.^25^ In a different population, we also found a 15% (95% confidence interval (CI): 6, 26) higher risk of obesity from late childhood through early adulthood.^18^ The association between birth by cesarean delivery and offspring obesity, and the increasing evidence that this risk may persist through adult life, raise the possibility that the adverse long-term offspring health effects of a cesarean section may extend to obesity-related chronic diseases.^26^ However, it is unclear whether there is a link between mode of delivery and systemic markers related to obesity and chronic disease, and published literature on the relationship between mode of birth and biochemical markers of chronic disease risk in the offspring remains scarce.^27–29^ To address this knowledge gap, we used data from Project Viva to assess differences in plasma levels of metabolic health and inflammation biomarkers during mid-childhood and early adolescence among children born by cesarean section compared to those born by vaginal delivery. We hypothesize that children born by cesarean section will show a more adverse profile of metabolic health and inflammation biomarkers compared to those born vaginally.

## Methods

### Study participants

**Fig. 1:**
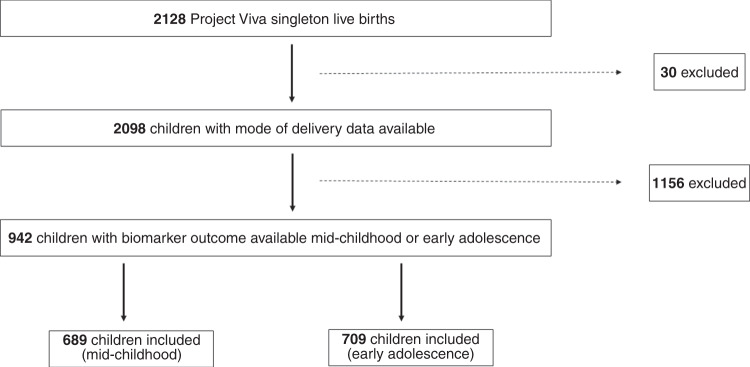
Study flowchart.

From April 1999 to July 2002, we enrolled participants into Project Viva, a longitudinal pre-birth cohort of mother–offspring pairs in eastern Massachusetts, USA. Institutional Review Boards of Harvard Pilgrim Health Care, Brigham and Women’s Hospital (BWH), and Beth Israel Deaconess Medical Center (BIDMC) approved study protocols,^30^ and all mothers provided written informed consent and children provided verbal assent at follow-up visits. Study population, enrollment, and follow-up procedures have been previously described in detail.^30^ Briefly, we recruited women attending their initial prenatal visit before 22 weeks gestational age at Atrius Harvard Vanguard Medical Associates, a multispecialty group practice. Eligibility criteria included fluency in English and singleton pregnancy. Most mothers gave birth at BWH or BIDMC. A trained research assistant conducted in-person study visits with the mother at the end of the first and second trimesters of pregnancy, and with both mother and child after delivery, during mid-childhood (median age 7 years) and early adolescence (median age 13 years). We collected data from multiple sources, including interviews/surveys, medical records, examinations, and biospecimens. Of the 2128 live singleton infants, 2098 had data on the mode of delivery (Fig. 1). This analysis includes 942 children with at least one available plasma metabolic health and inflammation biomarker level either measured during mid-childhood (*N* = 689) or early adolescence (*N* = 709). Rates of cesarean birth (22 vs. 25%) as well as participant characteristics, including maternal age, prepregnancy BMI, race/ethnicity, education, smoking, paternal BMI, and child sex, were similar between included and excluded participants (Supplemental Table 1).

### Mode of delivery

We obtained information about the mode of delivery from electronic hospital records. For each participant who had a cesarean section recorded on electronic birth logs, we reviewed the operative report to confirm cesarean section and to abstract the primary indication for operative delivery and the timing of cesarean section in relation to onset of labor. Using this information, we divided cesarean deliveries into unplanned cesarean sections preceded by labor and planned cesarean sections not preceded by labor. We defined an unplanned cesarean section as a delivery in which the operative report described a failed induction of labor, prolonged latent phase, prolonged active phase, arrest of dilation, “failure to progress,” arrest of descent in the second stage or failed operative vaginal delivery, “non-reassuring fetal heart rate tracing,” non-reassuring testing prompting immediate cesarean delivery, cord prolapse, or abruption.^21^ We defined planned cesarean sections as those for which participants did not undergo a trial of labor (elective repeat cesarean without trial of labor, malpresentation, placenta previa, suspected macrosomia, maternal request, or other indication precluding trial of labor).

### Metabolic health and inflammation biomarkers

Blood samples were collected by phlebotomists and stored within 24 h in liquid nitrogen freezers. We quantified an inflammatory marker [C-reactive protein (CRP), interleukin-6 (IL-6), adipokines (leptin, adiponectin), blood lipids (high-density lipoprotein cholesterol (HDL-C), triglycerides), and insulin resistance markers (glucose, insulin)] in plasma samples at the Clinical and Epidemiological Research Lab at Boston Children’s Hospital. The concentration of CRP was determined using an immunoturbidimetric assay on the Roche Cobas 6000 system (Roche Diagnostics, Indianapolis, IN). This high-sensitivity assay has a limit of detection of 0.03 mg/L. The mean intra-assay at concentrations of 0.91, 1.60, and 18.40 mg/L are 3.8%, 3.3%, and 1.9%, respectively. IL-6 was measured by an ultra-sensitive ELISA (enzyme-linked immunosorbent assay) assay from R&D Systems (Minneapolis, MN). The assay has a sensitivity of 0.094 pg/mL, and the mean intra-assay at concentrations of 0.49, 2.78 and 5.65 pg/mL are 9.6%, 7.2%, and 6.5%, respectively. Adiponectin was measured by an ELISA method from R&D Systems (Minneapolis, MN). The assay has a sensitivity of 0.25 ng/mL and the mean intra-assay at concentrations of 20.5, 74.4, and 157 ng/mL are 6.8%, 5.8%, and 6.9%, respectively. Leptin was measured by an ultra-sensitive ELISA assay, an enzymatically amplified “two-step” sandwich-type immunoassay (R&D Systems, Minneapolis, MN). The assay has a sensitivity of 7.8 pg/mL. The mean intra-assay at concentrations of 65.7, 146, and 581 pg/mL are 5.4%, 4.2%, and 3.5%, respectively. The determination of triglycerides and HDL-C concentrations was simultaneously performed on the Roche Cobas 6000 System using reagents and calibrators from Roche Diagnostics (Indianapolis, IN). Triglycerides at concentrations of 84.0 and 201.8 mg/dL were determined in the laboratory with a reproducibility of 1.8% (SD = 1.6 mg/dL) and 1.7% (SD = 3.5 mg/dL), respectively. We measured plasma glucose enzymatically and insulin using an electrochemiluminescence immunoassay (both Roche Diagnostics). We calculated insulin resistance in overnight blood samples using the homeostasis model (homeostatic model assessment of insulin resistance (HOMA-IR) = (insulin (µU/mL) × glucose (mmol/L)/22.5).^31^ To calculate the metabolic risk *z*-score in mid-childhood, we used the sex-specific internal *z*-scores averaging measures of waist circumference, triglycerides, inverted HDL-C, HOMA-IR, and systolic blood pressure. We calculated the metabolic risk *z*-score in early adolescence using age- and sex-specific external reference data.^32–34^

### Covariates

We collected sociodemographic and medical data through in-person interviews at enrollment, mid-childhood, and early adolescence visits, as well as yearly self-administered questionnaires, and hospital, and ambulatory medical records. Mothers reported their age, race/ethnicity, education, parity, prepregnancy weight and height, and paternal weight and height, and we calculated BMI as kg/m^2^ for both mothers and fathers. Also, we calculated gestational weight gain (GWG) by subtracting prepregnancy weight from the last prenatal weight. We obtained birth weight (BW) and delivery date from hospital medical records. We calculated gestational age (GA) at birth using the date of the last menstrual period. If the estimate of GA by second-trimester ultrasound assessment differed from the calculated GA by >10 days, we used the ultrasound dating. We determined sex-specific BW-for-GA *z*-scores using US reference data.^35^ We calculated a pubertal development score using a 5-item written pubertal development scale^36^ completed by the parents at the early adolescent visit. Items included on the boys’ pubertal development scale were voice deepening, body hair growth, facial hair growth, acne, and a growth spurt. Items included on the girls’ pubertal development scale were breast development, body hair growth, acne, growth spurt, and menarche. Response options and scores for each pubertal development scale item except menarche were: 1 point for “not yet started,” 2 points for “barely started,” 3 points for “definitely started,” and 4 points for “seems complete.” We coded menarche as 4 points if menarche was present and 1 point if not present. To assign a summary pubertal development score to each participant, we calculated the average score across all items.

### Statistical analyses

Participants’ characteristics as well as plasma metabolic health and inflammation biomarkers for the overall study population, as well as by mode of delivery, were presented using mean ± standard deviation (SD) or *n* (percentage). We initially defined the mode of delivery as a two-category variable: cesarean section and vaginal delivery. We then used data on timing and type of labor in relation to delivery and on planning of cesarean section and conducted separate analyses defining mode as a three-category [planned cesarean (all without trial of labor), unplanned cesarean (following spontaneous or induced labor), and vaginal delivery] variable. Plasma metabolic health and inflammation biomarker levels were natural log-transformed to approximate normality. We used multivariable linear regression models to estimate the association between mode of delivery and plasma metabolic health and inflammation biomarker levels in mid-childhood and early adolescence while adjusting for confounders. Confounders included covariates associated with both mode of delivery and plasma metabolic health and inflammation biomarker levels that were not in the causal pathway between both. Final models were adjusted for child age and sex at sample collection, maternal prepregnancy BMI, age, education, race/ethnicity, pregnancy smoking, GWG, BW/GA *z*-scores, and paternal BMI. We used stabilized inverse probability weights to account for censoring.^37^ To evaluate the robustness of the findings, we conducted a series of sensitivity analyses restricted to the first child per family (*N* = 923), excluding children whose mother experienced gestational diabetes or preeclampsia (*N* = 874), excluding macrosomic children (*N* = 773), excluding preterm births (*N* = 888), and simultaneously excluding children in these three groups (*N* = 671). We also conducted a sensitivity analysis restricted to fasting samples for insulin (*N* = 601), glucose (*N* = 610), and HOMA-IR (*N* = 587) outcomes in early adolescence. Results were reported as % difference (95% CI) [except for metabolic risk *z*-score, *β* (95% CI)]. All analyses were performed with SAS (version 9.4; SAS Institute Inc., Cary, NC). We chose not to employ classic multiple comparison correction techniques (i.e., Bonferroni), as these would be too conservative, given the correlated nature of the outcomes themselves.^38^

## Results

Maternal mean (SD) age was 31.9 (5.5) years and prepregnancy BMI was 25.1 (5.4) kg/m^2^ (Table 1). Of the 942 individuals with plasma metabolic health and inflammation biomarker data measured either during mid-childhood or early adolescence, 206 (22%) were born by cesarean section and 496 (53%) were males. Compared to women who delivered vaginally, women who delivered by cesarean section had higher prepregnancy BMI [means (SD) = 26.4 (6.5) vs. 24.7 (4.9)] and were more likely to have a college degree (72 vs. 66%). No other maternal, paternal, and offspring characteristics substantially differed by mode of delivery (Table 1).

**Table 1:** Characteristics of 942 mother–child pairs with either mild-childhood or early adolescence plasma metabolic health and inflammation biomarkers by mode of delivery (cesarean section vs. vaginal delivery) in Project Viva.

	Overall	Vaginal	Cesarean
	*N* = 942	*N* = 736 (78%)	*N* = 206 (22%)
Mother			
Age (years)	31.9 (5.5)	31.8 (5.7)	32.5 (5.0)
Prepregnancy BMI (kg/m^2^)	25.1 (5.4)	24.7 (4.9)	26.4 (6.5)
Total GWG (kg)	15.5 (5.4)	15.5 (5.2)	15.4 (6.0)
Race/ethnicity (%)			
Black	172 (18)	131 (18)	41 (20)
Hispanic	62 (7)	45 (6)	17 (8)
White	613 (65)	487 (67)	126 (61)
Other	90 (10)	69 (9)	21 (10)
College graduate (%)	629 (67)	482 (66)	147 (72)
Pregnancy smoking status (%)			
Never	650 (69)	501 (68)	149 (72)
Former	187 (20)	149 (20)	38 (18)
Current	102 (11)	83 (11)	19 (9)
Father’s BMI (kg/m^2^)	26.4 (4.0)	26.2 (3.9)	27.1 (4.1)
Child			
Female sex (%)	446 (47)	349 (47)	97 (47)
Birth weight for GA *z*-score	0.21 (0.99)	0.18 (0.96)	0.31 (1.08)
Age at mid-childhood visit (yearS)	8.0 (0.8)	8.0 (0.8)	7.9 (0.8)
Age at early adolescent visit (years)	13.1 (0.8)	13.1 (0.8)	13.1 (0.9)
Pubertal development score at early adolescent visit	2.5 (0.8)	2.5 (0.8)	2.5 (0.8)

Data are presented as mean (SD), unless stated otherwise.

Crude plasma concentrations of metabolic health and inflammation biomarkers, as well as metabolic risk *z*-score in mid-childhood, were similar in children born by cesarean section compared to those born vaginally (Table 2). Similarly, in adjusted models, there were no significant differences in plasma concentrations of metabolic health or inflammation biomarkers as well as metabolic risk *z*-score in mild-childhood by mode of delivery (Table 3).

**Table 2:** Plasma metabolic health and inflammation biomarker concentrations [mean (SD)] during mid-childhood and early adolescence by mode of delivery (cesarean section vs. vaginal delivery) in Project Viva.

	Overall	Vaginal	Cesarean	*P* value, vaginal vs. cesarean
Mid-childhood	*N* = 689	*N* = 534 (78%)	*N* = 155 (22%)	
Leptin (ng/mL)	6.1 (7.5)	6.3 (7.6)	5.3 (6.8)	0.17
Adiponectin (µg/mL)	15.6 (8.8)	15.8 (9.0)	14.9 (8.0)	0.30
Insulin (µU/mL)	7.9 (6.5)	8.0 (6.8)	7.4 (5.2)	0.25
Glucose (mg/dL)	94.5 (14.9)	94.7 (15.1)	93.7 (14.1)	0.50
HOMA-IR (U)	1.9 (1.8)	1.9 (1.9)	1.7 (1.5)	0.20
HDL-C (mg/dL)	57.0 (13.6)	57.3 (14.3)	56.3 (10.8)	0.38
Triglycerides (mg/dL)	57.9 (25.4)	58.2 (25.6)	56.6 (24.7)	0.51
IL-6 (pg/mL)	1.0 (1.4)	1.1 (1.5)	0.9 (1.0)	0.06
CRP (mg/L)	0.9 (2.9)	0.9 (2.8)	1.2 (3.4)	0.38
Metabolic risk *z*-score	0.00 (0.64)	0.00 (0.64)	−0.01 (0.62)	0.87
Early adolescence	*N* = 709	*N* = 549 (77%)	*N* = 160 (23%)	
Leptin (ng/mL)	12.0 (14.2)	11.6 (13.8)	13.1 (15.7)	0.30
Adiponectin (µg/mL)	6.4 (2.8)	6.5 (2.8)	5.8 (2.7)	0.01
Insulin (µU/mL)	16.1 (14.6)	15.4 (11.8)	18.5 (21.6)	0.09
Glucose (mg/dL)	92.5 (15.5)	92.5 (16.4)	92.2 (12.3)	0.81
HOMA-IR (U)	3.2 (2.3)	3.2 (2.2)	3.4 (2.6)	0.28
HDL-C (mg/dL)	55.0 (13.5)	55.4 (13.9)	53.6 (12.1)	0.12
Triglycerides (mg/dL)	70.9 (34.1)	71.2 (33.9)	70.0 (34.7)	0.70
IL-6 (pg/mL)	1.3 (1.6)	1.3 (1.7)	1.2 (1.4)	0.39
CRP (mg/L)	0.9 (2.1)	0.9 (2.3)	0.9 (1.7)	0.95
Metabolic risk *z*-score	−0.14 (0.46)	−0.16 (0.44)	−0.09 (0.53)	0.15

**Table 3:** Associations of the mode of delivery (cesarean section vs. vaginal delivery) with metabolic health and inflammation biomarkers during mid-childhood (*N* = 689) and early adolescence (*N* = 709) in Project Viva.

	Model 0	Model 1	Model 2
Mid-childhood			
Leptin (ng/mL)	−10.0 (−23.2, 5.5)	−13.4 (−26.1, 1.5)	−15.5 (−27.8, −1.1)
Adiponectin (µg/mL)	−5.2 (−15.3, 6.0)	−4.7 (−14.8, 6.7)	−5.3 (−15.4, 6.0)
Insulin (µU/mL)	−1.0 (−14.2, 14.2)	−6.5 (−19.0, 8.0)	−7.2 (−19.7, 7.2)
Glucose (mg/dL)	−1.7 (−4.6, 1.2)	−1.8 (−4.7, 1.2)	−1.8 (−4.7, 1.2)
HOMA-IR (U)	−6.2 (−19.7, 9.5)	−10.4 (−23.3, 4.8)	−11.5 (−24.3, 3.6)
HDL-C (mg/dL)	−0.4 (−4.9, 4.2)	−0.3 (−4.8, 4.3)	0.0 (−4.5, 4.7)
Triglycerides (mg/dL)	−1.3 (−8.8, 6.7)	−1.7 (−9.2, 6.4)	−1.8 (−9.3, 6.4)
IL-6 (pg/mL)	−10.0 (−23.8, 6.2)	−12.1 (−25.5, 3.7)	−12.4 (−25.8, 3.4)
CRP (mg/L)	14.3 (−16.4, 56.3)	4.2 (−23.6, 42.3)	0.0 (−26.7, 36.5)
Metabolic risk *z*-score	0.01 (−0.12, 0.14)	−0.04 (−0.17, 0.09)	−0.06 (−0.19, 0.07)
Early adolescence			
Leptin (ng/mL)	11.3 (−7.4, 33.7)	6.7 (−10.6, 27.3)	5.1 (−11.7, 25.2)
Adiponectin (µg/mL)	−12.6 (−19.3, −5.3)	−11.3 (−18.0, −4.0)	−11.3 (−18.1, −4.0)
Insulin (µU/mL)	12.4 (0.1, 26.1)	11.9 (−0.1, 25.3)	11.5 (−0.4, 25.0)
Glucose (mg/dL	−0.3 (−2.8, 2.2)	−0.7 (−3.1, 1.8)	−0.6 (−3.1, 1.9)
HOMA-IR (U)	11.3 (−0.8, 24.8)	9.9 (−1.6, 22.7)	9.1 (−2.3, 21.8)
HDL-C (mg/dL)	−3.2 (−7.5, 1.2)	−2.5 (−6.8, 2.0)	−2.2 (−6.5, 2.3)
Triglycerides (mg/dL)	−1.0 (−8.6, 7.2)	−1.5 (−9.1, 6.6)	−1.5 (−9.1, 6.7)
IL-6 (pg/mL)	−1.2 (−12.6, 11.6)	−3.0 (−14.1, 9.7)	−2.8 (−14.0, 9.9)
CRP (mg/L)	13.0 (−9.5, 41.2)	5.5 (−15.1, 31.2)	3.5 (−16.7, 28.5)
Metabolic risk *z*-score	0.09 (0.00, 0.19)	0.08 (−0.01, 0.17)	0.07 (−0.02, 0.16)

Plasma metabolic health and inflammation biomarkers were log-transformed and thus data are presented as % difference (95% CI) [except for metabolic risk *z*-score, *β* (95% CI)]. We used stabilized inverse probability weights to account for censoring.

Model 0: adjusted for child age and sex.

Model 1: Model 0 + maternal prepregnancy BMI, age, education, and race/ethnicity.

Model 2: Model 1 + total GWG, pregnancy smoking, BW/GA *z*-score, and father’s BMI.

*CRP* C-reactive protein, *HDL-C* high-density lipoprotein, *HOMA-IR* homeostatic model assessment of insulin resistance, *IL-6* interleukin-6.

However, in early adolescence, those born by cesarean section had lower adiponectin [% difference (95% CI) = −11.3 (−18.1, −4.0) µg/mL] compared to vaginal delivery. We also found a suggestion of higher insulin resistance reflected in associations with higher insulin levels (% difference (95% CI) = 11.5 (−0.40, 25.0) µU/mL) and higher HOMA-IR (% difference (95% CI) = 9.10 (−2.30, 21.8) U), neither of which was statistically significant. The associations of cesarean section with adiponectin and insulin levels in early adolescence were similar for planned and unplanned cesarean sections (Table 4), and persisted after adjustment for concurrent BMI-*z* (data not shown). There were no differences in other measured biomarkers as well as the metabolic risk *z*-score both in early adolescence between children born by cesarean section compared to vaginally (Table 3).

**Table 4:** Associations of mode of delivery (unplanned cesarean, planned vs. vaginal delivery) with metabolic health and inflammation biomarkers during mid-childhood (*N* = 689) and early adolescence (*N* = 709) in Project Viva.

	*N*	Model 0	Model 1	Model 2
Mid-childhood outcomes				
Leptin (ng/mL)				
Vaginal	481	1.0 (ref.)	1.0 (ref.)	1.0 (ref.)
CS unplanned/labor	90	−11.6 (−26.4, 6.2)	−13.2 (−27.7, 4.1)	−15.9 (−29.8, 0.8)
CS planned/no labor	42	−6.3 (−28.4, 22.7)	−14.1 (−34.6, 12.8)	−15.0 (−35.2, 11.6)
Adiponectin (µg/mL)				
Vaginal	481	1.0 (ref.)	1.0 (ref.)	1.0 (ref.)
CS unplanned/labor	90	−5.0 (−16.6, 8.2)	−4.9 (−16.5, 8.2)	−5.3 (−16.9, 7.8)
CS planned/no labor	42	−6.1 (−22.4, 13.6)	−4.6 (−21.4, 15.8)	−5.6 (−22.3, 14.7)
Insulin (µU/mL)				
Vaginal	474	1.0 (ref.)	1.0 (ref.)	1.0 (ref.)
CS unplanned/labor	89	−6.0 (−20.2, 10.7)	−9.1 (−22.8, 7.1)	−10.0 (−23.7, 6.0)
CS planned/no labor	41	25.8 (−1.6, 60.8)	13.4 (−11.7, 45.7)	13.9 (−11.5, 46.7)
Glucose (mg/dL)				
Vaginal	450	1.0 (ref.)	1.0 (ref.)	1.0 (ref.)
CS unplanned/labor	84	−3.4 (−6.7, 0.0)	−3.2 (−6.5, 0.2)	−3.3 (−6.6, 0.2)
CS planned/no labor	41	2.1 (−2.9, 7.3)	1.5 (−3.5, 6.9)	1.7 (−3.4, 7.0)
HOMA-IR (U)				
Vaginal	436	1.0 (ref.)	1.0 (ref.)	1.0 (ref.)
CS unplanned/labor	80	−12.4 (−26.8, 4.8)	−13.7 (−27.8, 3.2)	−14.9 (−28.9, 1.9)
CS planned/no labor	39	23.5 (−5.1, 60.6)	11.5 (−14.7, 45.7)	10.6 (−15.6, 44.9)
HDL-C (mg/dL)				
Vaginal	493	1.0 (ref.)	1.0 (ref.)	1.0 (ref.)
CS unplanned/labor	89	1.1 (−4.2, 6.7)	0.8 (−4.5, 6.4)	0.9 (−4.3, 6.5)
CS planned/no labor	45	−4.2 (−11.2, 3.4)	−3.4 (−10.6, 4.4)	−2.9 (−10.1, 5.0)
Triglycerides (mg/dL)				
Vaginal	492	1.0 (ref.)	1.0 (ref.)	1.0 (ref.)
CS unplanned/labor	89	0.1 (−8.7, 9.8)	−0.4 (−9.2, 9.2)	−0.5 (−9.3, 9.2)
CS planned/no labor	45	−4.2 (−15.9, 9.2)	−4.3 (−16.3, 9.4)	−4.4 (−16.5, 9.4)
IL-6 (pg/mL)				
Vaginal	481	1.0 (ref.)	1.0 (ref.)	1.0 (ref.)
CS unplanned/labor	90	−16.2 (−30.8, 1.5)	−16.3 (−30.8, 1.3)	−15.3 (−30.0, 2.5)
CS planned/no labor	42	6.1 (−19.9, 40.6)	−0.9 (−25.5, 31.7)	−4.3 (−28.1, 27.5)
CRP (mg/L)				
Vaginal	489	1.0 (ref.)	1.0 (ref.)	1.0 (ref.)
CS unplanned/labor	89	−0.9 (−31.6, 43.4)	−4.8 (−34.0, 37.2)	−7.5 (−35.8, 33.3)
CS planned/no labor	49	50.8 (−9.3,150.6)	24.1 (−25.6,106.8)	16.6 (−30.1, 94.5)
Metabolic risk *z*-score				
Vaginal	425	0.0 (ref.)	0.0 (ref.)	0.0 (ref.)
CS unplanned/labor	76	−0.06 (−0.21, 0.09)	−0.07 (−0.22, 0.08)	−0.10 (−0.25, 0.05)
CS planned/no labor	39	0.15 (−0.07, 0.37)	0.04 (−0.18, 0.26)	0.02 (−0.20, 0.24)
Early adolescent outcomes				
Leptin (ng/mL)				
Vaginal	534	1.0 (ref.)	1.0 (ref.)	1.0 (ref.)
CS unplanned/labor	102	4.9 (−15.4, 30.1)	1.2 (−17.6, 24.4)	−0.2 (−18.5, 22.3)
CS planned/no labor	49	25.6 (−7.4, 70.4)	19.1 (−11.2, 59.8)	17.8 (−11.8, 57.5)
Adiponectin (µg/mL)				
Vaginal	534	1.0 (ref.)	1.0 (ref.)	1.0 (ref.)
CS unplanned/labor	102	−13.2 (−21.0, −4.7)	−11.5 (−19.3, −3.0)	−11.5 (−19.3, −2.9)
CS planned/no labor	49	−10.9 (−22.0, 1.8)	−10.6 (−21.6, 1.9)	−10.7 (−21.7, 1.9)
Insulin (µU/mL)				
Vaginal	533	1.0 (ref.)	1.0 (ref.)	1.0 (ref.)
CS unplanned/labor	102	13.6 (−0.7, 30.0)	12.7 (−1.3, 28.6)	11.9 (−2.0, 27.7)
CS planned/no labor	49	8.9 (−10.1, 31.9)	9.6 (−9.2, 32.5)	10.2 (−8.8, 33.2)
Glucose (mg/dL)				
Vaginal	481	1.0 (ref.)	1.0 (ref.)	1.0 (ref.)
CS unplanned/labor	92	−0.9 (−3.7, 2.0)	−1.1 (−4.0, 1.8)	−1.2 (−4.0, 1.8)
CS planned/no labor	47	0.1 (−3.8, 4.2)	−0.4 (−4.3, 3.7)	−0.1 (−4.1, 4.0)
HOMA-IR (U)				
Vaginal	465	1.0 (ref.)	1.0 (ref.)	1.0 (ref.)
CS unplanned/labor	89	13.8 (−0.5, 30.3)	12.7 (−1.0, 28.3)	11.6 (−1.9, 27.0)
CS planned/no labor	44	4.2 (−13.8, 25.9)	2.5 (−14.5, 22.9)	2.3 (−14.7, 22.7)
HDL-C (mg/dL)				
Vaginal	534	1.0 (ref.)	1.0 (ref.)	1.0 (ref.)
CS unplanned/labor	102	−2.3 (−7.3, 3.0)	−1.8 (−6.8, 3.5)	−1.5 (−6.5, 3.7)
CS planned/no labor	49	−4.6 (−11.5, 2.8)	−3.3 (−10.3, 4.2)	−3.0 (−9.9, 4.6)
Triglycerides (mg/dL)				
Vaginal	534	1.0 (ref.)	1.0 (ref.)	1.0 (ref.)
CS unplanned/labor	102	−0.7 (−9.5, 8.9)	−0.4 (−9.2, 9.2)	−0.5 (−9.3, 9.1)
CS planned/no labor	49	−4.2 (−16.0, 9.3)	−6.7 (−18.2, 6.5)	−6.6 (−18.1, 6.6)
IL-6 (pg/mL)				
Vaginal	530	1.0 (ref.)	1.0 (ref.)	1.0 (ref.)
CS unplanned/labor	102	4.9 (−9.1, 20.9)	3.1 (−10.6, 18.9)	3.1 (−10.7, 18.9)
CS planned/no labor	49	−12.2 (−28.3, 7.6)	−13.9 (−29.7, 5.5)	−13.6 (−29.5, 6.0)
CRP (mg/L)				
Vaginal	519	1.0 (ref.)	1.0 (ref.)	1.0 (ref.)
CS unplanned/labor	98	12.7 (−13.1, 46.1)	6.6 (−17.3, 37.4)	4.5 (−18.8, 34.5)
CS planned/no labor	47	12.3 (−22.6, 63.0)	2.1 (−29.1, 47.0)	0.2 (−30.3, 44.1)
Metabolic risk *z*-score				
Vaginal	461	0.0 (ref.)	0.0 (ref.)	0.0 (ref.)
CS unplanned/labor	85	0.09 (−0.01, 0.20)	0.08 (−0.02, 0.19)	0.08 (−0.03, 0.18)
CS planned/no labor	43	0.08 (−0.07, 0.23)	0.05 (−0.10, 0.20)	0.04 (−0.10, 0.19)

Plasma metabolic health and inflammation biomarkers were log-transformed and thus data are presented as % difference (95% CI) [except for metabolic risk *z*-score, *β* (95% CI)]. We used stabilized inverse probability weights to account for censoring.

Model 0: adjusted for child age and sex.

Model 1: Model 0 + maternal prepregnancy BMI, age, education, and race/ethnicity.

Model 2: Model 1 + total GWG, pregnancy smoking, BW/GA *z*-score, and father’s BMI.

*CRP* C-reactive protein, *HDL-C* high-density lipoprotein, *HOMA-IR* homeostatic model assessment of insulin resistance, *IL-6* interleukin-6.

We then conducted a series of sensitivity analyses to evaluate the robustness of these findings. Estimates of the relation of the mode of delivery with adiponectin, insulin, and HOMA-IR were in the same direction and of approximately the same magnitude as in the main analysis, when we restricted analyses to the first child per family (Supplemental Table 2) and to fasting samples (Supplemental Table 3). Associations with adiponectin levels in early adolescence were also similar in analyses excluding children whose mothers experienced gestational diabetes or preeclampsia and preterm births (Supplemental Table 4). However, all associations were attenuated and no longer statistically significant when excluding macrosomic children, either separately or simultaneously with other preterm births and medically complicated pregnancies (Supplemental Table 4). Also when excluding macrosomic children, cesarean delivery was associated with lower leptin, HOMA-IR, and metabolic risk *z*-score in mid-childhood. However, selection bias is possible in this very selected subgroup.

## Discussion

In this prospective cohort study of mother–offspring pairs in eastern Massachusetts, we observed that, compared to children born vaginally, those born by cesarean section had lower adiponectin and higher insulin levels, as well as a suggestion of higher HOMA-IR, in early adolescence. The magnitude of these relations remained relatively constant across multiple sensitivity analyses aimed at evaluating the robustness of the results, although their statistical significance changed as sample size varied in these analyses. Our findings add to the expanding epidemiologic literature on long-term health risks for children associated with birth by cesarean delivery, but their clinical significance remains uncertain. Therefore, it is important that these relations be reevaluated in other studies

Epidemiological studies,^18–20^ including from Project Viva,^21^ as well as three meta-analyses,^22–24^ have shown a higher risk of childhood obesity in children born by cesarean section. While it is clear that childhood obesity is a risk factor for chronic diseases later in life,^39–42^ the epidemiologic literature on the mode of delivery with offspring biochemical markers of chronic diseases^27–29^ is scarce and inconclusive. A Brazilian cohort study found no differences in lipids, glucose, or markers of insulin resistance at age 23–25 years, despite higher BMI among young adults born by cesarean section.^28^ In contrast, a study among Danish young adults (age 20 years), birth by cesarean section was related to higher BMI, total cholesterol, low-density lipoprotein cholesterol, apolipoprotein B, and leptin levels.^29^ To our knowledge, this is the first study investigating the association between mode of delivery and systemic metabolic health and inflammation biomarkers in mid-childhood and early adolescence. Given the large differences in terms of socioeconomic factors, prevalence of childhood obesity, differences in age at outcome assessment, and frequency of birth by cesarean section across the three studies where this question has been addressed, it is critical that the relationship between mode of delivery and biomarkers of chronic disease risk is evaluated in additional prospective studies.

Some biological mechanisms have been proposed to explain the relation of cesarean section with childhood obesity that may also apply to the observed relations with adiponectin and insulin we observed in this study. Of note, multiple groups have reported differences in intestinal microbiota composition between infants delivered via cesarean vs. vaginally.^43–45^ Infants delivered via cesarean are less likely to be colonized with *Bacteroides* spp., and overall have lower community diversity.^46–49^ In adults, the ratio of *Firmicutes* to *Bacteroidetes* and gut microbial composition overall are related to obesity, weight, and body composition,^50–52^ and, importantly for our findings, insulin resistance.^53^ Although it is unknown whether differences in gut microbiota by mode of delivery observed in infants persist later in childhood or adolescence, such persistence could explain the observed relations. It is also important to consider why an association was observed in early adolescence but not earlier in childhood and only with adiponectin and insulin, but not with other markers of insulin resistance such as glucose or HOMA-IR. It is well documented that puberty is accompanied by a physiologic increase in insulin resistance that is, transient, characterized by a compensatory increase in insulin but no hyperglycemia, and independent of fat mass.^54^ This physiologic increase in insulin resistance would be expected to increase the variability between subjects in markers of insulin resistance in early adolescence relative to earlier in childhood resulting in greater power to identify differences. This increased variability can be observed in our data, particularly for insulin levels. Therefore, it is a possibility that a downstream consequence of cesarean section is an amplification of the physiologic insulin resistance of puberty. As our findings were of borderline statistical significance, despite robustness in direction and magnitude in sensitivity analysis, and since we did not account for multiple testing, a chance finding should be also considered as an equally plausible explanation for the observed relations. Clearly, new studies are necessary not only to corroborate or refute our findings but also to examine the underlying biological mechanisms underpinning these relations.

This study includes important strengths. First, we used a well-characterized prospective cohort with a large and detailed collection of covariates to reduce the possibility of residual confounding in the reported associations. Second, our study included objective measures of metabolic health and inflammation biomarkers obtained by highly trained staff using standardized protocols. Third, we used sophisticated statistical methods such as stabilized inverse probability weights to account for selection bias due to loss to follow-up, although similar cesarean section rates, plasma biomarker levels, and maternal-child characteristics were observed between included and excluded participants. Some limitations included self-reported maternal prepregnancy weight to calculate maternal BMI, which can cause measurement error; however, a correlation between self-reported and clinic prepregnancy weight in a subset of Project Viva participants was high (*r* = 0.99). Importantly, we did not assay glucose immediately, but instead used stored, frozen samples, which likely resulted in greater variability and this non-differential error would tend to bias results to the null. Also, generalizability is limited because there is a high proportion of white study participants who also had a relatively high level of education and income. In addition, it is important to consider the possibility that what we interpreted as a signal suggesting for increased insulin resistance in early adolescence among individuals born by cesarean section could also be a chance finding resulting from multiple testing. Finally, pubertal development information was collected by the parents using a questionnaire; thus, it is subject to measurement error. However, the questionnaire has been previously validated^36^ and the score has been strongly correlated with physician breast Tanner staging in girls^55^ and pubic hair staging in boys.^56^ Furthermore, the calculated pubertal score has been associated with BMI among children in Project Viva.^57^

In summary, we found that adolescents born by cesarean section have lower levels of adiponectin and higher levels of insulin, suggesting increased insulin resistance, relative to adolescents born by vaginal delivery. While these findings are intriguing, given the scarcity of data on this topic and its great potential public health impact, it is imperative that this question is addressed in independent studies to confirm or refute these findings, ideally in populations where additional details regarding the specific circumstances leading to the decision to perform a cesarean section can be examined.

## Supplementary information

Supplementary Information
