# Supplementary material for: Cesarean delivery and metabolic health and inflammation biomarkers during mid-childhood and early adolescence
Source: Pediatr Res. 2021 Apr 6;91(3):672–80. doi: 10.1038/s41390-021-01503-9 (PMC8492770; doi:10.1038/s41390-021-01503-9)
Supplement: Supplementary file 1 — Supplementary Information [file 41390_2021_1503_MOESM1_ESM.docx]

**Supplemental Table 1.** Characteristics of mother-child pairs included vs. excluded from the current analysis in Project Viva.

|  | **Total Viva Cohort** | **Included** | **Excluded** |
| --- | --- | --- | --- |
|  | N=2128 | N=942 | N=1186 |
| **Mother** |  |  |  |
| Mode of delivery, % |  |  |  |
| Vaginal | 1600 (76) | 736 (78) | 864 (75) |
| Cesarean | 498 (24) | 206 (22) | 292 (25) |
| Age, years | 31.8 ( 5.2) | 31.9 ( 5.5) | 31.7 ( 5.0) |
| Pre-pregnancy BMI, kg/m^2^ | 24.9 ( 5.5) | 25.1 ( 5.4) | 24.7 ( 5.6) |
| Total GWG, kg | 15.5 ( 5.7) | 15.5 ( 5.4) | 15.6 ( 5.9) |
| Race/ethnicity, % |  |  |  |
| Black | 348 (17) | 172 (18) | 176 (15) |
| Hispanic | 154 (7) | 62 (7) | 92 (8) |
| White | 1399 (66) | 613 (65) | 786 (67) |
| Other | 203 (10) | 90 (10) | 113 (10) |
| College graduate, % | 1360 (65) | 629 (67) | 731 (63) |
| Pregnancy smoking status, % |  |  |  |
| Never | 1443 (68) | 650 (69) | 793 (68) |
| Former | 398 (19) | 187 (20) | 211 (18) |
| Current | 266 (13) | 102 (11) | 164 (14) |
| Father's BMI, kg/m^2^ | 26.4 ( 4.1) | 26.4 ( 4.0) | 26.5 ( 4.2) |
| Female child sex, % | 1032 (48) | 446 (47) | 586 (49) |
| Birthweight for GA z-score | 0.17 (0.97) | 0.21 (0.99) | 0.15 (0.96) |

Data are presented as mean (SD) unless stated otherwise.

**Supplemental Table 2.** Associations of mode of delivery (cesarean section vs. vaginal delivery) with metabolic health and inflammation biomarkers during mid-childhood and early adolescence in Project Viva including the first child per family (N=923).

|  | **Model 0** | **Model 1** | **Model 2** |
| --- | --- | --- | --- |
| **Mid-childhood** |  |  |  |
| Leptin, ng/mL | -9.5 (-22.8, 6.0) | -13.1 (-25.9, 1.8) | -15.0 (-27.4, -0.5) |
| Adiponectin, µg/mL | -5.2 (-15.3, 6.0) | -4.6 (-14.8, 6.8) | -5.2 (-15.4, 6.2) |
| Insulin, µU/mL | -1.2 (-14.4, 14.0) | -6.9 (-19.4, 7.5) | -7.5 (-20.0, 6.9) |
| Glucose, mg/dL | -1.7 (-4.6, 1.2) | -1.9 (-4.7, 1.1) | -1.9 (-4.8, 1.1) |
| HOMA-IR, units | -6.5 (-20.0, 9.3) | -10.9 (-23.7, 4.2) | -11.9 (-24.7, 3.1) |
| HDL-C, mg/dL | -0.5 (-4.9, 4.2) | -0.4 (-4.9, 4.3) | 0.0 (-4.5, 4.7) |
| Triglycerides, mg/dL | -1.6 (-9.0, 6.4) | -1.9 (-9.4, 6.2) | -2.0 (-9.4, 6.2) |
| IL6, pg/mL | -10.0 (-23.7, 6.3) | -12.1 (-25.6, 3.8) | -12.6 (-26.0, 3.2) |
| CRP, mg/L | 14.8 (-16.1, 57.1) | 4.4 (-23.6, 42.7) | 0.2 (-26.6, 36.8) |
| Metabolic risk z-score | 0.01 (-0.12, 0.14) | -0.04 (-0.17, 0.09) | -0.06 (-0.19, 0.07) |
| **Early adolescence** |  |  |  |
| Leptin, ng/mL | 11.5 (-7.3, 34.1) | 6.5 (-10.8, 27.2) | 4.9 (-11.9, 25.0) |
| Adiponectin, µg/mL | -12.3 (-19.1, -4.9) | -11.0 (-17.7, -3.6) | -11.0 (-17.8, -3.7) |
| Insulin, µU/mL | 12.4 (0.1, 26.2) | 11.7 (-0.4, 25.2) | 11.4 (-0.6, 24.9) |
| Glucose, mg/dL | -0.3 (-2.7, 2.2) | -0.6 (-3.1, 1.9) | -0.6 (-3.0, 2.0) |
| HOMA-IR, units | 11.3 (-0.8, 25.0) | 9.6 (-1.9, 22.5) | 8.9 (-2.5, 21.7) |
| HDL-C, mg/dL | -3.1 (-7.4, 1.3) | -2.4 (-6.7, 2.1) | -2.1 (-6.4, 2.4) |
| Triglycerides, mg/dL | -0.8 (-8.5, 7.4) | -1.4 (-9.0, 6.8) | -1.4 (-9.0, 6.9) |
| IL6, pg/mL | -1.4 (-12.7, 11.4) | -3.2 (-14.4, 9.4) | -3.0 (-14.2, 9.7) |
| CRP, mg/L | 14.2 (-8.4, 42.4) | 6.0 (-14.6, 31.6) | 4.2 (-16.0, 29.1) |
| Metabolic risk z-score | 0.09 (0.00, 0.19) | 0.07 (-0.02, 0.16) | 0.07 (-0.02, 0.16) |

Plasma metabolic health and inflammation biomarkers were log-transformed thus data are presented as % difference (95% CI) [except for metabolic risk z-score, β (95% CI)]. We used stabilized inverse probability weights to account for censoring.

Model 0. Adjusted for child age and sex

Model 1. Model 0 + maternal pre-pregnancy BMI, age, education and race/ethnicity.

Model 2. Model 1 + total GWG, pregnancy smoking, BW/GA z-score and father's BMI.

Abbreviations: C-Reactive Protein, CRP; High-Density Lipoprotein, HDL-C; Homeostatic Model Assessment of Insulin Resistance, HOMA-IR; Interleukin-6, IL6.

**Supplemental Table 3.** Associations of mode of delivery (cesarean section vs. vaginal delivery) with insulin (N=601), glucose (N=610) and HOMA-IR (N=587) outcomes in early adolescence in Project Viva restricted to fasting samples.

|  | **Model 0** | **Model 1** | | **Model 2** | |  |
| --- | --- | --- | --- | --- | --- | --- |
| **Early adolescence** |  | |  | |  | |
| Insulin, µU/mL | 10.5 (-0.9, 23.2) | | 9.5 (-1.5, 21.6) | | 8.7 (-2.1, 20.7) | |
| Glucose, mg/dL | -0.2 (-2.6, 2.2) | | -0.5 (-2.9, 1.9) | | -0.5 (-2.9, 1.9) | |
| HOMA-IR, units | 10.7 (-1.3, 24.2) | | 9.1 (-2.3, 21.9) | | 8.3 (-3.1, 20.9) | |

Plasma metabolic health biomarkers were log-transformed thus data are presented as % difference (95% CI). We used stabilized inverse probability weights to account for censoring.

Model 0. Adjusted for child age and sex

Model 1. Model 0 + maternal pre-pregnancy BMI, age, education and race/ethnicity.

Model 2. Model 1 + total GWG, pregnancy smoking, BW/GA z-score and father's BMI.

Abbreviations: Homeostatic Model Assessment of Insulin Resistance, HOMA-IR.

**Supplemental Table 4.** Associations of mode of delivery (cesarean section vs. vaginal delivery) with metabolic health and inflammation biomarkers during mid-childhood and early adolescence in Project Viva excluding A) children whose mother experienced gestational diabetes or preeclampsia (N=874), B) macrosomic children (N=773), C) preterm births (N=888) and D) simultaneously excluding children in these three groups (N=671).

|  | **A** | **B** | **C** | **D** |
| --- | --- | --- | --- | --- |
| **Mid-childhood** |  |  |  |  |
| Leptin, ng/mL | -14.3 (-27.7, 1.5) | -17.7 (-31.3, -1.5) | -16.9 (-29.6, -1.8) | -19.2 (-34.3, -0.7) |
| Adiponectin, µg/mL | -3.5 (-14.6, 9.1) | -4.0 (-15.6, 9.2) | -6.1 (-16.6, 5.7) | -0.8 (-14.5, 15.1) |
| Insulin, µU/mL | -4.9 (-18.3, 10.7) | -11.3 (-24.8, 4.6) | -8.8 (-21.3, 5.6) | -14.6 (-28.6, 2.3) |
| Glucose, mg/dL | -1.3 (-4.5, 1.9) | -3.1 (-6.3, 0.2) | -2.0 (-5.0, 1.1) | -3.5 (-7.0, 0.2) |
| HOMA-IR, units | -8.4 (-22.4, 8.3) | -17.6 (-31.0, -1.6) | -14.4 (-27.0, 0.3) | -22.0 (-35.7, -5.4) |
| HDL-C, mg/dL | -0.4 (-5.1, 4.5) | 1.2 (-4.0, 6.7) | -0.2 (-4.9, 4.7) | 1.3 (-4.5, 7.5) |
| Triglycerides, mg/dL | -1.2 (-9.0, 7.3) | -3.5 (-12.0, 5.8) | -2.6 (-10.4, 5.9) | -3.6 (-12.9, 6.5) |
| IL6, pg/mL | -12.2 (-26.6, 5.0) | -10.1 (-25.5, 8.5) | -8.8 (-23.5, 8.7) | -8.2 (-26.1, 14.0) |
| CRP, mg/L | 1.4 (-27.2, 41.3) | 5.3 (-25.8, 49.4) | -3.6 (-30.5, 33.6) | 2.4 (-31.1, 52.3) |
| Metabolic risk z-score | -0.04 (-0.18, 0.09) | -0.12 (-0.27, 0.02) | -0.10 (-0.23, 0.04) | -0.17 (-0.33,-0.01) |
| **Early adolescence** |  |  |  |  |
| Leptin, ng/mL | 6.5 (-11.2, 27.8) | 7.7 (-11.6, 31.2) | 4.2 (-13.4, 25.4) | 5.5 (-15.5, 31.5) |
| Adiponectin, µg/mL | -11.5 (-18.6, -3.9) | -8.6 (-16.4, 0.0) | -10.7 (-17.9, -3.0) | -5.8 (-14.7, 4.1) |
| Insulin, µU/mL | 12.2 (-0.4, 26.5) | 11.0 (-2.4, 26.2) | 12.6 (-0.2, 27.1) | 13.0 (-2.2, 30.7) |
| Glucose, mg/dL | -0.3 (-2.9, 2.3) | -0.4 (-3.2, 2.4) | -0.6 (-3.2, 2.1) | -0.4 (-3.5, 2.9) |
| HOMA-IR, units | 8.9 (-3.0, 22.2) | 7.5 (-5.2, 21.8) | 8.2 (-3.8, 21.6) | 5.1 (-8.5, 20.8) |
| HDL-C, mg/dL | -1.8 (-6.4, 2.9) | -0.8 (-5.7, 4.2) | -1.8 (-6.3, 3.0) | 0.4 (-5.1, 6.2) |
| Triglycerides, mg/dL | -1.9 (-9.8, 6.6) | 0.3 (-8.3, 9.7) | -2.8 (-10.6, 5.6) | -1.9 (-11.1, 8.3) |
| IL6, pg/mL | -4.2 (-15.9, 9.1) | -1.1 (-14.5, 14.3) | -7.4 (-18.4, 5.0) | -9.1 (-22.4, 6.4) |
| CRP, mg/L | 3.2 (-18.0, 29.7) | 5.8 (-17.4, 35.6) | -2.3 (-22.1, 22.6) | -0.9 (-24.7, 30.5) |
| Metabolic risk z-score | 0.06 (-0.03, 0.15) | 0.07 (-0.03, 0.17) | 0.05 (-0.05, 0.14) | 0.01 (-0.10, 0.12) |

Plasma biomarker health outcomes were log-transformed thus data are presented as % difference (95% CI) [except for metabolic risk z-score, β (95% CI)]. We used stabilized inverse probability weights to account for censoring. Models are adjusted for child age and sex, maternal pre-pregnancy BMI, age, education and race/ethnicity as well as total GWG, pregnancy smoking, BW/GA z-score and father's BMI. Abbreviations: C-Reactive Protein, CRP; High-Density Lipoprotein, HDL-C; Homeostatic Model Assessment of Insulin Resistance, HOMA-IR; Interleukin-6, IL6.
